# Multidimensional employment trajectories and dynamic links with mental health: Evidence from the UK Household Longitudinal Study

**DOI:** 10.5271/sjweh.4193

**Published:** 2025-01-01

**Authors:** Rebeka Balogh, Sylvie Gadeyne, Christophe Vanroelen, Chris Warhurst

**Affiliations:** 1 School of Health & Wellbeing, University of Glasgow, Glasgow, UK.; 2 Brussels Institute for Social and Population Studies (BRISPO), Department of Sociology, Vrije Universiteit Brussel, Brussels, Belgium.; 3 Institute for Employment Research, University of Warwick, Coventry, UK.

**Keywords:** employment quality, job quality, precarious employment, sequence analysis

## Abstract

**Objectives:**

Low-quality and precarious employment have been associated with adverse mental health and wellbeing. More evidence is needed on how the quality of employment trajectories – including transitions in and out of unemployment, inactivity, and employment of varying quality – are associated with individuals’ mental health over time. This paper aimed to derive a typology of multidimensional employment trajectories and assess associations with mental health in the UK.

**Methods:**

Data from waves 1–9 of the UK Household Longitudinal Study were used (2009–2019). Individuals aged 30–40 at baseline were included (N=1603). Using multichannel sequence and clustering analyses, we derived a typology of employment trajectories across employment statuses and four employment quality indicators. We assessed associations with subsequent psychological distress, accounting for baseline mental health. Changes in average General Health Questionnaire scores are described.

**Results:**

A typology of five trajectory clusters highlighted stable and secure and precarious/low-quality trajectories for both men and women. Women who reported being economically inactive at most waves had higher odds of experiencing psychological distress than did women in ‘standard’ trajectories, regardless of baseline mental health. Women’s scores of psychological distress in the ‘precarious’ group on average increased along their trajectories characterized by instability and transitions in/out of unemployment, before a move into employment. Men who likely moved in and out of unemployment and economic inactivity, with low probability of paid employment, reported increased psychological distress at the end of follow-up. This may partly be due to pre-existing mental ill-health.

**Conclusion:**

This paper shows the importance of high-quality employment for individuals’ mental health over time. Researchers need to consider dynamic associations between employment quality and mental health across the life-course.

It has long been recognized that labor market flexibilization (1–3) poses a health risk to workers (4). Recently, it has been suggested that flexible labor markets’ adverse health effect is in fact best captured by focusing on the multiple adverse conditions of employment that make up precarious employment (5). Precarious employment represents a multidimensional concept characterized by instability, lack of protection, low control, insufficient income and, overall, multiple insecurities (3, 6–8). It stands in contrast to what has been described as the “Standard Employment Relationship” (SER), an employment form thought to represent stability, security and overall high employment quality (9–11). Precarious employment and SER sit on the spectrum of employment quality – comprising the many employment conditions and relations – representing, respectively, an accumulation of disadvantageous and advantageous features (10, 11). Moreover, on the same spectrum, multiple other ‘types’ of employment arrangements can be found, offering various levels of employment quality to the worker as products of different modes of destandardisation affecting the SER (10, 11). Employment quality focuses on objective features of the employment arrangement and as such is distinct from job satisfaction and subjective feelings of job insecurity (12). While a broad multidimensional approach with a wider range of employment quality indicators is preferable to best capture these employment types and potential health associations (11), shorter versions of the concept with a limited set of measures have also been used, focusing on income/pay, multiple job-holding, working time, and employment stability (13, 14).

## Employment (quality) across the life-course

Empirical evidence to date has underlined employment quality (and low employment quality, precarious employment, in particular) as a social determinant of mental health outcomes (10, 15). The mechanisms assumed to link precarious employment to adverse (mental) health are related to disadvantage through material deprivation, higher exposure to other work-related physical and psychosocial health risks, and/or ‘direct’ effects coming from psychosocial stressors such as insecurity, feelings of injustice and powerlessness related to a situation of precariousness (10, 16). As much of the current evidence is of a cross-sectional nature, calls for more longitudinal studies into the health associations of precarious employment have been made (16, 17). First, such studies would increase our understanding of the instability and volatility associated with precarious employment (18). Mapping out trajectories and monitoring eventual changes in employment quality are therefore indispensable. This allows us to better understand not only the configurations of employment quality but also how dynamics, changes in employment quality and transitions from and to employment statuses other than waged-employment make up the overall quality of individuals’ employment trajectories, including more precarious, low-quality, trajectories (13, 14, 19). Trajectories characterized by lower attachment to the labor market, precariousness and instability have indeed shown adverse mental health associations compared to stable trajectories of full-time employment and decent wage, and such analyses increasingly consider the multidimensional nature of employment trajectories (13, 14, 19, 20). Second, longitudinal inquiries can shed light on how health (dis)advantage is shaped by potentially accumulating risk factors along employment trajectories (21–23). Specifically, precarious and low-quality employment may adversely impact individuals’ health and poor health may create further disadvantages by limiting individuals’ employment or earnings opportunities (24), which could then in turn lead to (further) low-quality trajectories and reinforce health inequalities across the employment quality spectrum (16). Therefore, looking at changes in mental health alongside employment status and employment quality can give us a more accurate picture of how the two interact over individuals’ life-course.

## Gender differences in (the quality of) employment trajectories

Women’s and men’s employment trajectories can differ due to the way the gendered distribution of unpaid work responsibilities impact men’s and women’s careers and life-courses differently (25). There is also a concern that women’s health may be more susceptible to precarious employment than men’s (26). A recent cross-national investigation, however, suggested that although the prevalence of poor mental health and of precarious employment was higher among women than men, the association between the two was more pronounced among men (27). Nevertheless, to better understand links between women’s and men’s employment trajectories and their mental health, more gender-sensitive, including gender-stratified, evidence is needed (28).

## Employment quality in the United Kingdom

In recent years, concerns have been voiced regarding a widening health gap and a growth in insecure jobs in England (29), as well as underemployment across the United Kingdom (UK) (30). The prevalence of high-quality employment may in fact be lower in the UK compared to (some) countries of the European Union, while the prevalence of precarious employment with lower pay and more likely involuntary part-time work has been shown to be higher (11). A specific study looking at employment quality in the UK found that those in low employment quality forms of employment (precarious and part-time forms characterized by lower pay and several other adverse features) were indeed associated with low mental well-being, but not with higher odds of psychological distress, in comparison to those in high employment quality (31). This study, however, focused only those in waged-employment, not considering those who may have been out of a job at the time of the survey (32). Thus, further investigations focusing on the quality of employment trajectories and psychological distress in the UK are warranted.

## Objectives

In sum, further research is needed to understand how low-quality employment forms and employment trajectories – including potential transitions in and out of employment – are related to psychological distress over time in the UK. This paper aims to fill this gap by deriving a typology of multidimensional employment trajectories and providing a more in-depth description of changes in mental health.

## Methods

### Data and study sample

Data for our analyses is derived from the first nine waves of the UK Household Longitudinal Study (also called Understanding Society), a longitudinal survey of households carried out in the UK since 2009 (33). Understanding Society collects information on a variety of household- and individual-level characteristics, including employment and self-reported health indicators. An analysis of Understanding Society’s main representative sample noted that attrition over time was notable and more likely among those with poor general health, but that sample weights successfully correct for differential attrition (34). Our analysis was restricted to individuals with complete information on employment status and the four employment quality dimensions, as well as their mental health over the nine waves, and who were aged 30–40 years at the time of the first wave (N=1603). The University of Essex Ethics Committee approved Understanding Society’s data collection (by letter dated 6 July 2007 for Waves 1 and 2; 17 December 2010 for Waves 3 to 5; 20 August 2013 for Waves 6 to 8; and 4 October 2016 for Waves 9-11 (main survey).

### Measures

*Employment quality indicators.* Information on employment status was elicited in all waves and we distinguished between (i) employees, (ii) the self-employed, (iii) the unemployed, and (iv) those who were economically inactive or other (retired, on maternity leave, family care, student/apprentice, in training, long-term sick or with a disability or working unpaid in family business). For those who indicated that they were presently employed, we assessed their employment quality in the following four dimensions: employment stability, pay, multiple jobholding, and working time. This represents a ‘condensed’ version of the set of employment quality indicators (13) that have been successfully applied to the same dataset in a prior study, and which can be found in all waves of the survey (31).

### Mental health outcome

Psychological distress was measured using the General Health Questionnaire (GHQ) 12-item questionnaire (35). Psychological distress was represented by a score of 3–12 using the ‘caseness’ scoring, with sensitivity analyses conducted using a score of 4–12 (35–37). Changes in mental health were described using the GHQ Likert score (range 0–36, with higher scores indicating potentially worse mental health; see also below). Further sensitivity analyses are also run using GHQ Likert score as a continuous outcome in regression modelling.

### Statistical analyses

A typology of employment trajectories was constructed using multichannel sequence analysis (MCSA) for men and women separately. Both sequence (19) and latent class (14) analyses can be used to derive multidimensional employment trajectory types; an investigation showed the two techniques can yield similar results for life-course trajectories (38). We chose to first model trajectories and then to apply a clustering technique next through MCSA as this way were able to easily model any changes between employment statuses and did not have to make any assumptions about measurement (in)variance through the 10-year study period (see reference 39).

MCSA enables the modelling of sequences within multiple interrelated domains, and, by that, extends traditional sequence analysis that typically would focus on sequences/trajectories in one domain/dimension at a time (19, 40, 41). Information on individuals’ employment status and employment quality in the four dimensions (described above) from each of the nine waves was used to reconstruct their employment trajectories. MCSA and clustering enabled us to describe them and distil ideal-types of employment trajectories across the four dimensions by grouping similar trajectories together (42).

In practical terms, the analysis involved a number of steps. First, we defined the sequence objects, calculated the dissimilarities [which would often, but not always, be the ‘cost’ of transforming one sequence into another (42)] and then used a clustering algorithm to partition the observations into groups which share similarities but which are as different from other clusters as possible (43, 44). In this paper, we chose ‘longest common subsequence’ as a dissimilarity metric to calculate the distances between trajectories, as this stresses similarities and differences between trajectories when it comes to the sequencing (ie, in what order individuals experience employment statuses and aspects of employment quality) and the time spent in a state (how long an individual is exposed to a certain aspect of employment) (42). Sensitivity analyses with another dissimilarity metric were also conducted, showing very similar results (42). Ward hierarchical clustering was then applied to the dissimilarity matrix and cluster solutions are examined from a theoretical perspective and looking at the values of average silhouette width (ASW), a partition quality indicator (42, 44, 45). The sequences were described and examined, including assessing mean times spent in different employment status/quality characteristic, and associations between the domains were assessed prior to building the typology (42, 46).

Associations between the typology and psychological distress measured at wave 9 (the end of follow-up) were then evaluated by fitting logistic regressions. Models were adjusted by age, and we also included partnership status, ethnic background (dichotomized as White British or not White British), and highest educational attainment as covariates (36). The last model included an adjustment for baseline psychological distress (16). In addition, descriptive statistics were provided to examine the characteristics of each of the clusters over time. To examine changes in mental health over time, mean GHQ (Likert) scores were plotted for each employment trajectory cluster across the nine waves under study (47). Analyses were conducted separately by gender. Analyses were run using R (48), including with the TraMineR package (49). Some code for analyses and graphs was developed by Raab & Struffolino (42).

## Results

### Descriptive statistics

Table S1 in the supplementary material (www.sjweh.fi/article/4193) includes a description of the study sample. Women and men on average shared similar background characteristics. Shannon’s entropy values (supplementary table S2) indicate that there is decreasing heterogeneity with regards to employment stability and working hours over the study period (42, 50). The mean time spent in each state (representing the number of waves each state was reported) for men and women differed accordingly with regards to (long and marginal part-time) hours, economic inactivity, and relative pay (supplementary tables S3–6). As supplementary tables S7–10 also show, there were important differences between men and women with regards to the states experienced and not experienced (42) over the nine waves. Whereas over half of women at one point reported being economically inactive/other, this proportion was more negligible among men. Nearly half of men in our sample reported working >48 hours a week in total, whereas over 80% of women never reported working long hours.

### Trajectory typology

Having examined the cluster solutions with different numbers of clusters, and their corresponding ASW values, a 5-cluster typology was chosen to best describe men’s and women’s multidimensional employment trajectories (ASW values shown in supplementary figures S1 and S2). The ASW values were relatively low and pointed to the most parsimonious, 2-cluster solution, as is common (42). Typologies with more clusters were, however, deemed preferable as they revealed more information. The 6-cluster solution contained groups with very low numbers while the 4-cluster solution among women grouped the economically inactive and the precarious groups together, while, among men, it did not distinguish between ‘high effort’ and ‘high-income’. Therefore, the 5-cluster typology was selected for further analyses for both genders.

**Figure 1:**
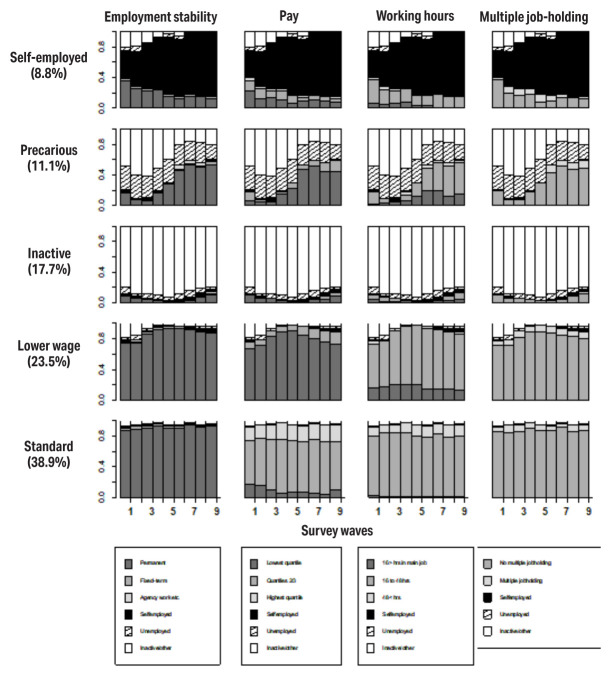
State distribution plots for five trajectory clusters, **women**.

**Figure 2:**
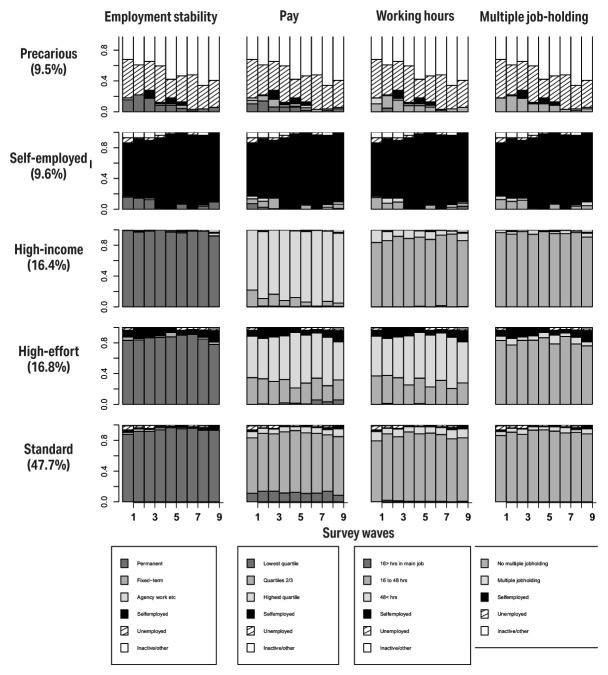
State distribution plots for five trajectory clusters, **men**.

These gender-specific typologies are shown in figures 1 and 2 for women and men, respectively. The state distribution graphs represent the proportion of individuals in a given survey wave who reported the specific employment statuses and employment quality characteristics, and not individual trajectories along the x axis (42).

Among both men and women, a large group reporting earnings in the 2^nd^ to 3^rd^ quartile, stable employment with some probability of long working hours could be observed, making up around 50% and 40% of the samples, respectively (labelled as ‘standard’ groups). Among both genders, a relatively small group which reported being self-employed at most waves of the survey could be observed (‘self-employed’).

In terms of gender-specific clusters, a sizable female cluster called ‘lower wage’ crystallized; women in this group quite consistently reported low earnings, stemming in part from their lower weekly working hours. Nearly 1 in 5 women had a trajectory characterized by near-constant reporting of economic inactivity (for brevity, we use the term ‘Inactive’ to describe this trajectory) over the nine waves of the survey. Among men, a ‘high effort’ (31) group could also be observed with frequent reporting of working >48 hours a week and often receiving high earnings in compensation. The ‘high-income’ group, however, consistently reported high earnings but without the long hours.

A ‘precarious’ group was derived among both men and women, though they were characterized by different features. A common feature in both was a high level of volatility observed from one wave to another, frequent spells of unemployment and inactivity. Among men, however, only a low proportion of individuals in the precarious group reported being employed across any of the survey waves; among women this percentage was higher, and some level of transitioning into employment could be observed.

### Characteristics of the clusters

Cross-tabulations with constant and time-varying characteristics are shown in table 1. Among women, the lowest proportion of ‘no partner in the household’ at wave 9 was observed in the precarious and inactive trajectories. The average hours spent on housework (see reference 51) was lowest among the standard and self-employed groups throughout, and higher in the precarious, inactive and lower wage trajectories. In the precarious trajectory, 60% of men had no partner living with them at the end of the study period. This revealed stark differences with men in other trajectories.

**Table 1a:** Characteristics of trajectory types - **women**. [SD=standard deviation]

Characteristic		Standard			Lower wage			Precarious			Inactive			Self-employed	
		%	Mean (SD)		%	Mean (SD)		%	Mean (SD)		%	Mean (SD)		%	Mean (SD)
Ethnic background															
	White British	82			86			86			78			83	
	Not White British	18			14			14			22			17	
Age at baseline			35.2 (3.2)			35.8 (3.2)			34.2 (3.2)			35.2 (3.1)			35.5 (3.0)
Partnership status, wave 9															
	No partner in household	22			31			41			38			30	
	Partner in household	78			69			59			62			70	
Hours spent on housework/week, wave 2			11 (6)			17 (10)			19 (14)			20 (13)			13 (9)
Hours spent on housework/week, wave 4			10 (7)			15 (8)			18 (11)			20 (15)			11 (7)
Hours spent on housework/week, wave 6			10 (7)			14 (9)			15 (12)			19 (14)			10 (6)
Hours spent on housework/week, wave 8			10 (7)			14 (10)			17 (12)			19 (13)			11 (7)

**Table 1b:** Characteristics of trajectory types - **men**. [SD=standard deviation]

Characteristic		Standard			High effort			Self-employed			High income			Precarious	
		%	Mean (SD)		%	Mean (SD)		%	Mean (SD)		%	Mean (SD)		%	Mean (SD)
Ethnic background															
	White British	81			83			87			82			89	
	Not White British	19			17			13			18			11	
Age at baseline			35.1 (3.3)			35.4 (3.1)			35.9 (3.0)			35.9 (3.2)			35.9 (3.0)
Partnership status, wave 9															
	No partner in household	21			16			23			7.2			60	
	Partner in household	79			84			77			93			40	
Hours spent on housework/week, wave 2			5.7 (4.8)			6.1 (5.2)			4.8 (4.7)			5.6 (4.2)			8.3 (6.0)
Hours spent on housework/week, wave 4			6.6 (5.0)			5.6 (5.2)			5.1 (4.1)			5.2 (3.8)			9.8 (9.5)
Hours spent on housework/week, wave 6			5.8 (4.6)			5.4 (4.1)			5.1 (4.7)			5.7 (3.8)			6.8 (5.9)
Hours spent on housework/week, wave 8			6.6 (5.0)			5.9 (4.2)			5.2 (3.8)			6.1 (4.7)			9.2 (7.6)

### Associations with psychological distress

**Figure 3:**
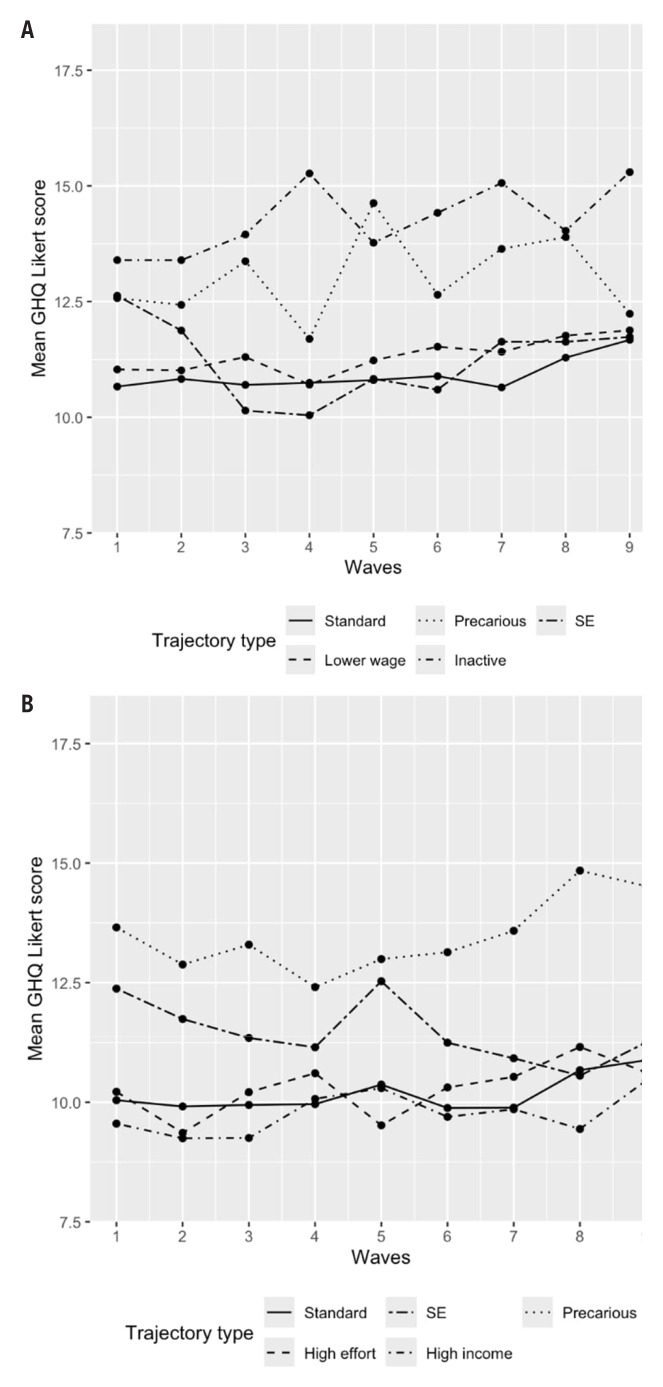
Average GHQ score (Likert) over time by trajectory type for a) women and b) men. Higher scores indicate higher levels of psychological distress. SE=self-employed.

Results of the regression analyses are presented in table 2. Mean GHQ scores are plotted for the trajectories in figure 3, and mean GHQ scores with 95% confidence intervals (CI) for select trajectories are plotted in supplementary figures S5 and S6 to ensure visual clarity.

**Table 2:** Associations between employment trajectory type and psychological distress at the end of the follow-up. [OR=odds ratio; CI=confidence interval.]

		N ^a^	Model 1 ^b^		Model 2 ^c^		Model 3 ^d^
			OR (95%CI)		OR (95%CI)		OR (95%CI)
Women							
	Standard	316	1.00		1.00		1.00
	Precarious	90	1.04 (0.55–1.95)		0.87 (0.46–1.65)		0.83 (0.45–1.53)
	Self-employed	72	0.89 (0.47–1.67)		0.8 (0.42–1.52)		0.7 (0.37–1.32)
	Inactive	144	2.29*** (1.49–3.52)		2.11** (1.35–3.3)		1.93** (1.22–3.07)
	Lower wage	191	0.85 (0.57–1.29)		0.76 (0.49–1.18)		0.74 (0.48–1.15)
	Observations ^e^		994		994		994
Men							
	Standard	372	1.00		1.00		1.00
	Precarious	74	2.43* (1.01–5.88)		3.19* (1.18–8.65)		2.54 (0.88–7.34)
	High effort	131	1.05 (0.57–1.93)		0.93 (0.5–1.73)		0.9 (0.47–1.72)
	Self-employed	75	1.59 (0.75–3.34)		1.6 (0.74–3.44)		1.47 (0.74–2.94)
	High income	128	0.97 (0.52–1.83)		0.89 (0.46–1.71)		0.88 (0.45–1.71)
	Observations ^e^		609		609		609

^a^ Weighted frequencies. ^b^ Adjusted by age. ^c^ Adjusted by age, partnership status, ethnic background, and educational attainment. ^d^ Adjusted by age, partnership status, ethnic background, educational attainment and baseline psychological distress. ^e^ Unweighted. *P<0.05. **P<0.01. ***P<0.001.

Regression analysis shows that women with an inactive trajectory had 2-fold increased odds of experiencing psychological distress compared to women with a standard trajectory and this associations persisted after adjusting for educational attainment, ethnic background, and partnership status at the end of the follow-up (see table 1). This association remained robust to adjusting for psychological distress at baseline. None of the other trajectories were related to increased psychological distress at the end of the follow-up, however. Sensitivity analyses reveal that a different cut-off point on the GHQ does not meaningfully alter these estimates (supplementary table S11). Further analyses also showed that the same patterning emerged for the trajectory typologies when examining the GHQ Likert scores as a continuous outcome (supplementary table S12). Examining mean GHQ Likert scores over time (see figure 3) as well as in psychological distress, however, suggest that at certain time points, individuals in the precarious group had comparable average mental health scores and prevalence of psychological distress to those in the inactive cluster, but that the gap between the standard and the precarious group reduced towards the end of the follow-up. Regression analyses and assessments of distress prevalence also confirm this association (data not shown). This improvement in mental health also coincides with an increased transition to paid (albeit lower quality) employment, as shown in the state distribution graphs.

Men in precarious trajectories had around 2.5-fold increased odds of reporting psychological distress at the end of the follow-up compared to their counterparts in the standard trajectory (table 2). This association, however, was explained by the inclusion of psychological distress at baseline. Sensitivity analyses showed that taking a higher (3/4) cut-off on the GHQ-12 (supplementary table S13) results in a statistically significant difference with the standard trajectory. Examining the trends over time along the precarious trajectory suggested some improvements in mental health over the study period with deterioration towards the end of the follow-up (figure 3).

## Discussion

This paper highlighted and examined a typology of multidimensional employment trajectories for men and women across a decade in the UK. Over nine survey waves, we considered transitions in and out of unemployment, economic inactivity, as well as employment of varying quality in terms of pay, working time, stability and multiple job-holding. Women’s and men’s trajectories were in several ways quite divergent which again underlined the need for gender-sensitive analyses (25, 27, 28).

Our analysis overall suggested that unstable trajectories or trajectories characterized by a prolonged exposure to economic inactivity (which may be due to health reasons) and unemployment are overall associated with poorer mental health subsequently, echoing findings from other country contexts (13, 14, 19, 20). Women who mostly reported being economically inactive or other across the survey waves under study reported higher odds of experiencing psychological distress even after accounting for baseline distress. This finding is in line with prior research from the UK showing a mental health disadvantage for those outside of the labor force (52, 53). Employment status was found to be an important mechanism in explaining class (52) and educational (53) inequalities in mental health in Great Britain/England, with the unemployed, long-term ill and looking after home/family reporting poorer psychological wellbeing and mental health than the employed (52, 53). In terms of a more general health indicator, evidence also suggests that rising levels of economic inactivity due to illness and disability likely contributed to increasing prevalence of poor self-rated health in the UK between 1978 and 2004 (54). We emphasize that we distinguished between the unemployed and economically inactive based on self-reports and did not consider whether individuals were actively seeking work (55), and note that some individuals may be outside the labor force due to mental ill-health (52). Our findings, nevertheless, show that a notable proportion of individuals were likely outside of the labor force for prolonged periods of time (women), or experienced spells of inactivity and/or unemployment over the study period (men), and this had long-term mental health associations compared to a ‘core’ group with apparent stable and higher-quality employment. Among men, adjusting for baseline psychological distress seemed to explain the association between the precarious trajectory and subsequent psychological distress. A number of factors may explain this finding, including pre-existing mental ill-health leading to ‘health selection’ out of employment (16), and the possibility that prior employment history had already exerted negative influence on mental health (21), as well as small sample size. For a more elevated psychological distress, however, inequalities were observed between the precarious and standard group regardless of individuals’ mental health in 2009/2011.

One important contribution of the paper is that it demonstrated the need for a more dynamic perspective on how mental health and employment trajectories and employment quality interacts over the life course. Descriptive analysis suggested there may be variations across time in mental health. Analyses coming from the British Household Panel Study indeed showed that individuals’ mental health can fluctuate (56). Sequence analysis literature recommends that in multivariate analyses, covariates from the start or the end of the study period be chosen as those from the ‘middle’ of trajectories may in fact be ‘endogenous’ to the typology (42, p. 110). At the same time, analyses such as ours reminds us that (good) health in fact fosters individuals’ ability and opportunity to engage in paid employment (of a certain quality) (57). We also may need to further nuance our perspective on ‘health selection’ and ‘health causation’ (58) and examine how in fact poor-quality employment trajectories may lead to adverse mental health which in turn, again, may affect the subsequent quality of employment or lead to unemployment (16). Indeed, it has been suggested that health selection and health causation are not in fact mutually exclusive mechanisms (59). For those in more precarious forms of employment, adverse health may in fact have more severe consequences and may reinforce or exacerbate already existing health inequalities across the labor market (24). However, future more refined analyses should further reveal how the incidence of a (mental) health condition is related to subsequent employment quality and how that in turn affects mental health later on.

The limitations of the study need to be stressed. First, we restricted the analysis on individuals who had complete information over the nine waves of the survey under study, and as a result, our sample size was limited, potentially affecting the robustness of our analyses. Our restriction also meant that our sample perhaps represented an overall healthier population (60). We also had a rather limited set of employment quality indicators for which information was collected in every wave. Individuals were interviewed annually, and so there may have been changes in their employment status or quality in-between waves which are not reflected in this analysis. Finally, individuals’ exposure to low employment quality or unemployment prior to baseline may have already negatively affected their mental health (21) or influenced their subsequent employment trajectories – an aspect we were not able to consider in this paper.

Nevertheless, there are several strengths to this paper. One of them lies in its longitudinal nature and its ability to account for psychological distress at baseline, which may have influenced individuals’ propensity to become or remain out of employment as well as their employment quality. We also were able to describe (possible) changes in mental health over a period of time. Considering men’s and women’s trajectories not only highlighted divergent trajectories but also showed that precarious careers may in fact bear different characteristics for men and women.

### Concluding remarks

While a large part of the UK’s population in their 30s and 40s reported a trajectory characterized by well-paid and stable employment each year between 2009 and 2019, a significant proportion of the population were either repeatedly economically inactive or reported unstable and precarious trajectories with movements in/out of unemployment or lower-quality employment. It is noted that repeated economic inactivity among women was associated with psychological distress at the end of the follow-up, irrespective of psychological distress at baseline. Among men, movements in and out of unemployment and inactivity and lower-quality employment, were associated with poorer mental health, and this may have been (in part) due to prior poor mental health limiting employment opportunities. Men’s and women’s mental health, however, evolved along their employment trajectory, particularly for women in precarious trajectories who showed a mental health disadvantage at certain points in time under study. Overall, this study highlights the need to consider the cumulative health impacts of multidimensional employment trajectories. Findings should be tested with larger samples.

## Supplementary material

Supplementary material
