# Supplementary material for: Multidimensional employment trajectories and dynamic links with mental health: Evidence from the UK Household Longitudinal Study
Source: Scand J Work Environ Health. 2024 Dec 30;51(1):26–37. doi: 10.5271/sjweh.4193 (PMC11697615; doi:10.5271/sjweh.4193)
Supplement: Supplementary material [file SJWEH-51-26-S001.pdf]

Supplementary material to Multidimensional employment trajectories and dynamic links with mental health. Evidence from the UK Household Longitudinal Study

Table S1: Sample description. n (%); Mean (SD)

**Characteristic**	**men**, N = 779	**women**, N = 812
Ethnic background		
White British	645 (83%)	673 (83%)
Not White British	134 (17%)	139 (17%)
Age at baseline	35.4 (3.2)	35.3 (3.2)

Table S2: Overview of Shannon's entropy

Wave	Employment stability	Pay	Working hours	Multiple job-holding
wave 1	0.60	0.91	0.76	0.67
wave 4	0.56	0.90	0.74	0.65
wave 7	0.53	0.90	0.73	0.65

Table S3: Mean time spent in states, employment stability

State	Mean, men	SD, men	Proportion of time, men	Mean, women	SD, women	Proportion of time, women
PERM	6.9	3.2	0.76	5.6	3.6	0.62
FT	0.1	0.5	0.01	0.2	0.6	0.02
TAW	0.1	0.3	0.01	0.1	0.4	0.01
SE	1	2.4	0.11	0.6	1.9	0.07
UE	0.5	1.4	0.05	0.5	1.2	0.05
IA/O	0.5	1.6	0.05	2.1	2.9	0.23

PERM= permanent employment, FT=fixed-term, TAW=temporary agency etc., SE=self-employed, UE=unemployed, IA/O=inactive/other

Table S4: Mean time spent in states, pay

State	Mean, men	SD, men	Proportion of time, men	Mean, women	SD, women	Proportion of time, women
Q1	0.6	1.7	0.07	2.4	3.1	0.27
Q2/3	3.9	3.6	0.43	2.7	3.2	0.3
Q4	2.5	3.4	0.28	0.7	2	0.08
SE	1	2.4	0.11	0.6	1.9	0.07
UE	0.5	1.4	0.05	0.5	1.2	0.05
IA/O	0.5	1.6	0.05	2.1	2.9	0.23

Q1= Bottom quartile, Q2/Q3=Quartiles 2/3, Q4=Top quartile, SE=self-employed, UE=unemployed, IA/O=inactive/other

Table S5: Mean time spent in states, working hours

State	Mean, men	SD, men	Proportion of time, men	Mean, women	SD, women	Proportion of time, women
16>	0.1	0.3	0.01	0.6	1.6	0.07
16to48	5.5	3.4	0.61	4.7	3.6	0.52
48<	1.5	2.3	0.16	0.5	1.5	0.06
SE	1	2.4	0.11	0.6	1.9	0.07
UE	0.5	1.4	0.05	0.5	1.2	0.05
IA/O	0.5	1.6	0.05	2.1	2.9	0.23

16> = <16 hours in main job, 16to48= >16 hours in main job and <48 hours in total, 48< = over 48 hours in total, SE=Self-employed, UE=unemployed, IA/O=inactive/other

Table S6: Mean time spent in states, multiple job-holding

State	Mean, men	SD, men	Proportion of time, men	Mean, women	SD, women	Proportion of time, women
No MJH	6.6	3.3	0.74	5.3	3.5	0.59
MJH	0.4	1.2	0.05	0.5	1.3	0.06
SE	1	2.4	0.11	0.6	1.9	0.07
UE	0.5	1.4	0.05	0.5	1.2	0.05
IA/O	0.5	1.6	0.05	2.1	2.9	0.23

No MJH=Not multiple job-holder, MJH=multiple job-holder, SE=Self-employed, UE=unemployed, IA/O=inactive/other

Table S7: Proportion of men and women who never reported a certain employment stability state

id	PERM	FT	TAW	SE	UE	IA/O
Men	0.13	0.93	0.95	0.78	0.83	0.87
Women	0.21	0.89	0.92	0.86	0.80	0.48

PERM= permanent employment, FT=fixed-term, TAW=temporary agency etc., SE=Self-employed, UE=unemployed, IA/O=inactive/other

Table S8: Proportion of men and women who never reported a certain pay state

id	Q1	Q2/3	Q4	SE	UE	IA/O
Men	0.82	0.33	0.54	0.78	0.83	0.87
Women	0.49	0.48	0.82	0.86	0.80	0.48

Q1= Bottom quartile, Q2/Q3=Quartiles 2/3, Q4=Top quartile, SE=Self-employed, UE=unemployed, IA/O=inactive/other

Table S9: Proportion of men and women who never reported a certain working hours state

id	16>	16to48	48<	SE	UE	IA/O
Men	0.96	0.17	0.55	0.78	0.83	0.87
Women	0.79	0.25	0.81	0.86	0.80	0.48

16> = <16 hours in main job, 16to48= >16 hours in main job and <48 hours in total, 48< = over 48 hours in total, SE=self-employed, UE=unemployed, IA/O=inactive/other

Table S10: Proportion of men and women who never reported a certain multiple job-holding state

id	No MJH	MJH	SE	UE	IA/O
Men	0.14	0.82	0.78	0.83	0.87
Women	0.20	0.78	0.86	0.80	0.48

No MJH=Not multiple job-holder, MJH=multiple job-holder, SE=self-employed, UE=unemployed, IA/O=inactive/other

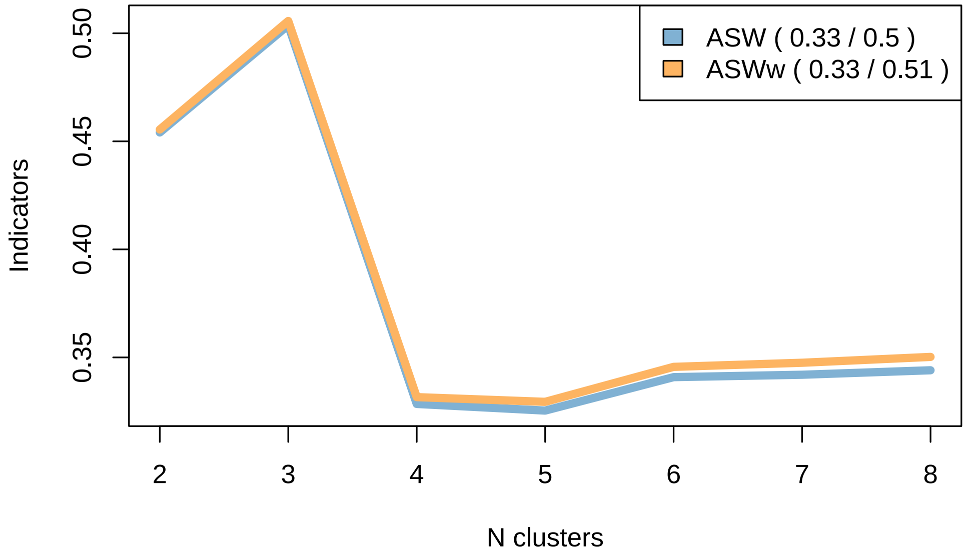

Figure S1: Average Silhouette Width per k-cluster solution, women

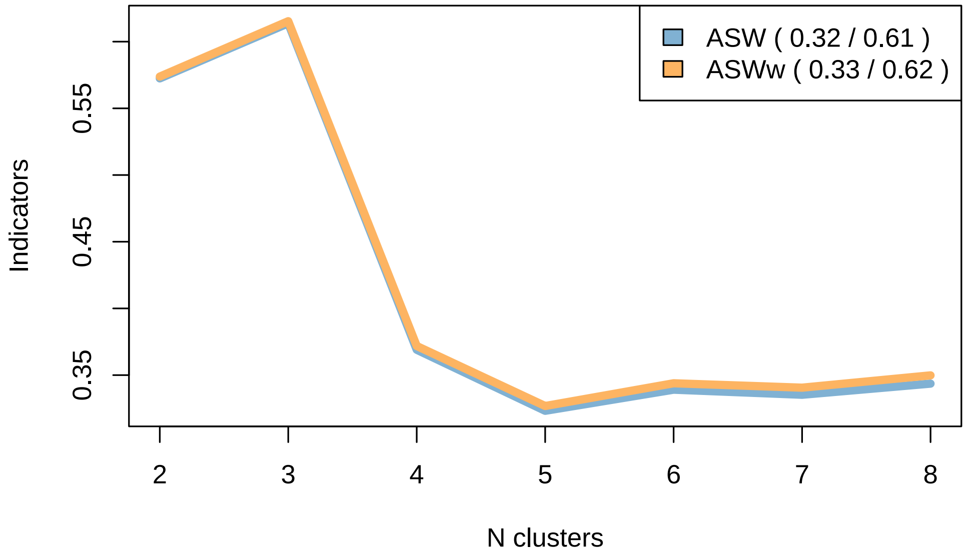

Figure S2: Average Silhouette Width per k-cluster solution, men

Table S11: Associations between employment trajectory and subsequent psychological distress among women. Odds ratios (and 95% confidence intervals). Sensitivity analyses with 3/4 cut-off. Ref: Standard trajectory

	Dependent variable:		
	GHQ-12 caseness - 3/4		
	Model 1	Model 2	Model 3
Lower wage	0.94	0.81	0.78
	(0.61, 1.44)	(0.51, 1.29)	(0.49, 1.25)
Precarious	1.32	1.09	1.03
	(0.68, 2.55)	(0.57, 2.10)	(0.54, 1.94)
Inactive	2.54***	2.31***	2.02**
	(1.64, 3.93)	(1.46, 3.66)	(1.24, 3.29)
Self-employed	1.08	0.98	0.84
	(0.55, 2.11)	(0.50, 1.94)	(0.43, 1.61)
Observations	994	994	994

Note: *p<0.05; **p<0.01; ***p<0.001
Model 1 is adjusted by age, Model 2 is adjusted by age, partnership status, ethnic background, and educational attainment, Model 3 is additionally adjusted by baseline psychological distress

Table S12: Associations between employment trajectory and GHQ-12 Likert score among women. Coefficients (and 95% confidence intervals). Ref: Standard trajectory

	Dependent variable:		
	GHQ-12 Likert score		
	Model 1	Model 2	Model 3
Lower wage	0.17	−0.18	−0.21
	(−0.83, 1.16)	(−1.26, 0.90)	(−1.24, 0.82)
Precarious	0.62	0.19	−0.43
	(−1.24, 2.49)	(−1.61, 1.99)	(−2.14, 1.27)
Inactive	3.63***	3.32***	2.41**
	(1.89, 5.37)	(1.65, 4.99)	(0.64, 4.17)
Self-employed	0.04	−0.22	−0.82
	(−1.34, 1.43)	(−1.60, 1.16)	(−2.06, 0.42)
Observations	994	994	994

Note: *p<0.05; **p<0.01; ***p<0.001

Model 1 is adjusted by age, Model 2 is adjusted by age, partnership status, ethnic background, and educational attainment, Model 3 is additionally adjusted by baseline GHQ Likert score

Table S13: Associations between employment trajectory and subsequent psychological distress among men. Odds ratios (and 95% confidence intervals). Sensitivity analyses with 3/4 cut-off. Ref: Standard trajectory

	Dependent variable:		
	GHQ-12 caseness - 3/4		
	Model 1	Model 2	Model 3
High effort	0.98	0.86	0.90
	(0.51, 1.88)	(0.44, 1.68)	(0.46, 1.79)
Self-employed	1.52	1.51	1.40
	(0.67, 3.45)	(0.65, 3.52)	(0.65, 3.01)
High income	0.88	0.77	0.76
	(0.45, 1.71)	(0.39, 1.54)	(0.38, 1.50)
Precarious	2.47	3.53*	3.66*
	(0.99, 6.16)	(1.23, 10.13)	(1.18, 11.38)
Observations	609	609	609

Note: *p<0.05; **p<0.01; ***p<0.001
Model 1 is adjusted by age, Model 2 is adjusted by age, partnership status, ethnic background, and educational attainment, Model 3 is additionally adjusted by baseline psychological distress

Table S14: Associations between employment trajectory and GHQ-12 Likert score among men. Coefficients (and 95% confidence intervals). Ref: Standard trajectory

	Dependent variable:		
	GHQ-12 Likert score		
	Model 1	Model 2	Model 3
High effort	−0.26	−0.50	−0.52
	(−1.50, 0.98)	(−1.74, 0.74)	(−1.71, 0.68)
Self-employed	0.37	0.30	−0.35
	(−0.92, 1.65)	(−0.98, 1.59)	(−1.51, 0.81)
High income	−0.43	−0.66	−0.51
	(−1.50, 0.64)	(−1.79, 0.48)	(−1.56, 0.55)
Precarious	3.63*	3.95**	3.08*
	(0.82, 6.44)	(1.49, 6.42)	(0.64, 5.52)
Observations	609	609	609
Note:	*p<0.05; **p<0.01; ***p<0.001		
	Model 1 is adjusted by age, Model 2 is adjusted by age, partnership status, ethnic background, and educational attainment, Model 3 is additionally adjusted by baseline GHQ Likert score		

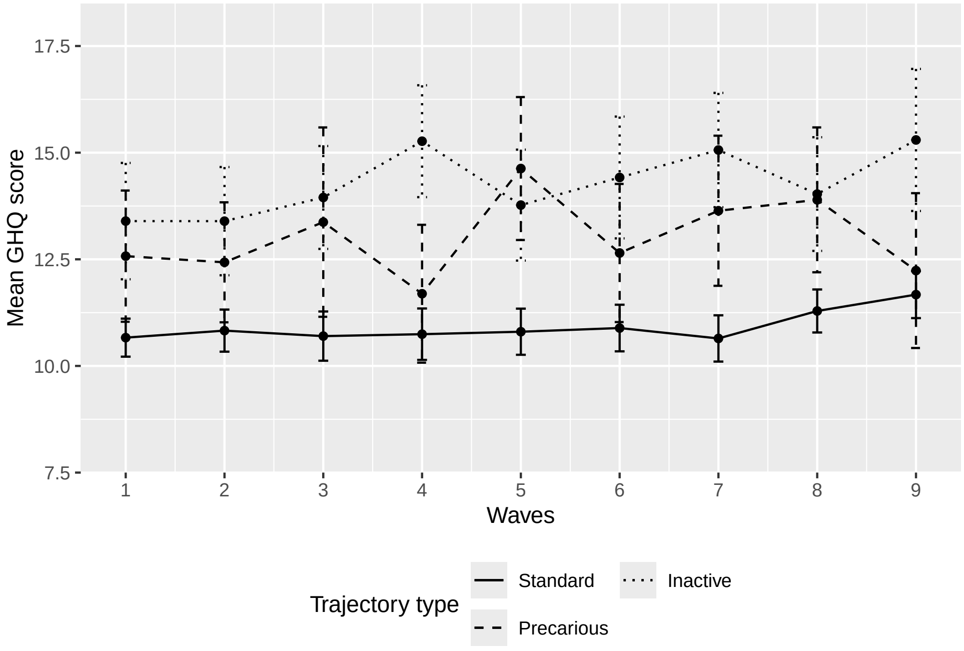

Figure S3: Average GHQ score (Likert) over time for select trajectories with 95% CI, women

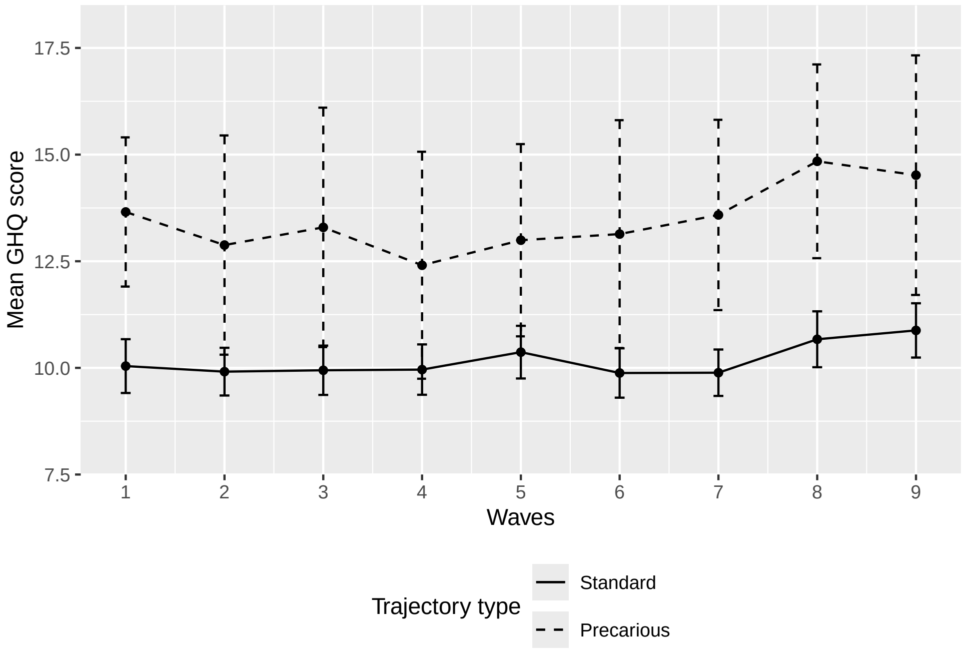

Figure S4: Average GHQ score (Likert) over time for select trajectories with 95% CI, men
